# Fault Diagnosis for the Heat Exchanger of the Aircraft Environmental Control System Based on the Strong Tracking Filter

**DOI:** 10.1371/journal.pone.0122829

**Published:** 2015-03-30

**Authors:** Jian Ma, Chen Lu, Hongmei Liu

**Affiliations:** 1 School of Reliability and Systems Engineering, Beihang University, Beijing, China; 2 Science & Technology on Reliability & Environmental Engineering Laboratory, Beijing, China; University of Washington, UNITED STATES

## Abstract

The aircraft environmental control system (ECS) is a critical aircraft system, which provides the appropriate environmental conditions to ensure the safe transport of air passengers and equipment. The functionality and reliability of ECS have received increasing attention in recent years. The heat exchanger is a particularly significant component of the ECS, because its failure decreases the system’s efficiency, which can lead to catastrophic consequences. Fault diagnosis of the heat exchanger is necessary to prevent risks. However, two problems hinder the implementation of the heat exchanger fault diagnosis in practice. First, the actual measured parameter of the heat exchanger cannot effectively reflect the fault occurrence, whereas the heat exchanger faults are usually depicted by utilizing the corresponding fault-related state parameters that cannot be measured directly. Second, both the traditional Extended Kalman Filter (EKF) and the EKF-based Double Model Filter have certain disadvantages, such as sensitivity to modeling errors and difficulties in selection of initialization values. To solve the aforementioned problems, this paper presents a fault-related parameter adaptive estimation method based on strong tracking filter (STF) and Modified Bayes classification algorithm for fault detection and failure mode classification of the heat exchanger, respectively. Heat exchanger fault simulation is conducted to generate fault data, through which the proposed methods are validated. The results demonstrate that the proposed methods are capable of providing accurate, stable, and rapid fault diagnosis of the heat exchanger.

## Introduction

An environmental control system (ECS) plays a critical function in aircrafts and guarantees the safety and comfort of passengers and crew as well as the normal operation of electronic equipment on board under different flight conditions. The ECS provides air supply, thermal control, and cabin pressurization for the crew and passengers. Avionics cooling is an essential component of the ECS of an aircraft. Specifically, as the power density of electronic equipment on board continuously increases, thus releasing large amounts of heat, the electronic equipment may fail with the increased operation temperature if the released heat is not diverted in time [1]. Therefore, ensuring the reliable operation of the ECS is crucial. However, with the increasing complexity of the ECS, the occurrence of faults has become inevitable. For this reason, it is essential to diagnose ECS faults effectively and carry out maintenance in a timely manner.

The heat exchanger is the primary and most commonly used component of the ECS, and its reliability directly determines the efficiency of the ECS. Thus, the accurate and effective fault diagnosis of the heat exchanger guarantees reliable ECS operation. Consequently, the heat exchanger is chosen as the object of the fault diagnosis method in this study.

Publications on the aircraft heat exchanger fault diagnosis is relative limited. The existing research on heat exchangers for aircrafts is mainly focused on system design, structure optimization, and simulation [2–4]. However, only limited research has been conducted on the fault diagnosis of the ECS, particularly that of the heat exchanger.

As discussed in reference [5], the common methods of heat exchanger diagnosis are summarized as follows:

1. Examination of the heat transfer coefficient (or the effectiveness).
2. Simultaneous observations of the pressure drops and mass flow rates.
3. Temperature measurements, e.g. (*t*_*h*.*in*_ – *t*_*h*.*out*_)/(*t*_*h*.*in*_ – *t*_*h*.*out*_)_*design*_.
4. Ultrasonic or electrical measurements.
5. Weighing of heat exchanger plates.
6. Model based method: modelling the heat exchanger and monitoring any discrepancy between model estimations and actual measurements.

To be very accurate, the prior four methods require that the heat exchanger presents successive steady states, i.e. the inlet temperatures and flows must be stable for a period long enough to be able to compute or measure the values of interest, or are local. The fifth method requires the operation process stops when diagnosing faults. The majority of these methods are far too restrictive or costly. Fortunately, the model-based methods are expected to avoid the existing problems.

The most commonly used model based method for heat exchanger is EKF [5–7], which is used for detecting fouling. The extended Kalman filter (EKF) is used to jointly solve the nonlinear estimation of the model parameters and states of the heat exchanger. Although EKF can realize the dynamic parameter estimation, a single EKF in a nonlinear system may lose its tracking ability when the process states change abruptly; this occurs once the process has reached a steady state and does not exhibit good ability for either the normal process (the steady state) or the fault process (the transient state). A fault diagnosis algorithm based on the double model filter (DMF) has been presented to deal with this problem [8]. That work used two EKF filters to track the respective processes in the DMF. However, both the EKF and the DMF (based on the EKF) have certain disadvantages, such as sensitivity to modeling errors and difficulties in the selection of initialization value.

To overcome the aforementioned disadvantages, the strong tracking filter (STF) [9] is employed to improve the existing EKF method and estimate the heat exchanger fault parameters. A Modified Bayes (MB) [10] classification algorithm is also developed to realize the fault detection and failure modes classification of the heat exchanger. Heat exchanger faults simulation is conducted to generate fault data, through which the proposed methods are validated. The results demonstrate that the proposed approaches are capable of providing accurate, stable, and rapid fault diagnosis of the heat exchanger.

This paper is organized as follows. Section II introduces the work principle of the ECS and the heat exchanger, and then presents the dynamic model of the heat exchanger. Fault information on the heat exchanger is introduced in Section III. The heat exchanger model introduced in Section II is modified for state estimation in Section IV. Section V describes an efficient estimating approach, namely, the STF approach. The experimental results are reported in Section VI. Conclusions are drawn in the final section.

## Background on Heat Exchanger

### Work Principle of the ECS and Heat Exchanger

**Fig 1:**
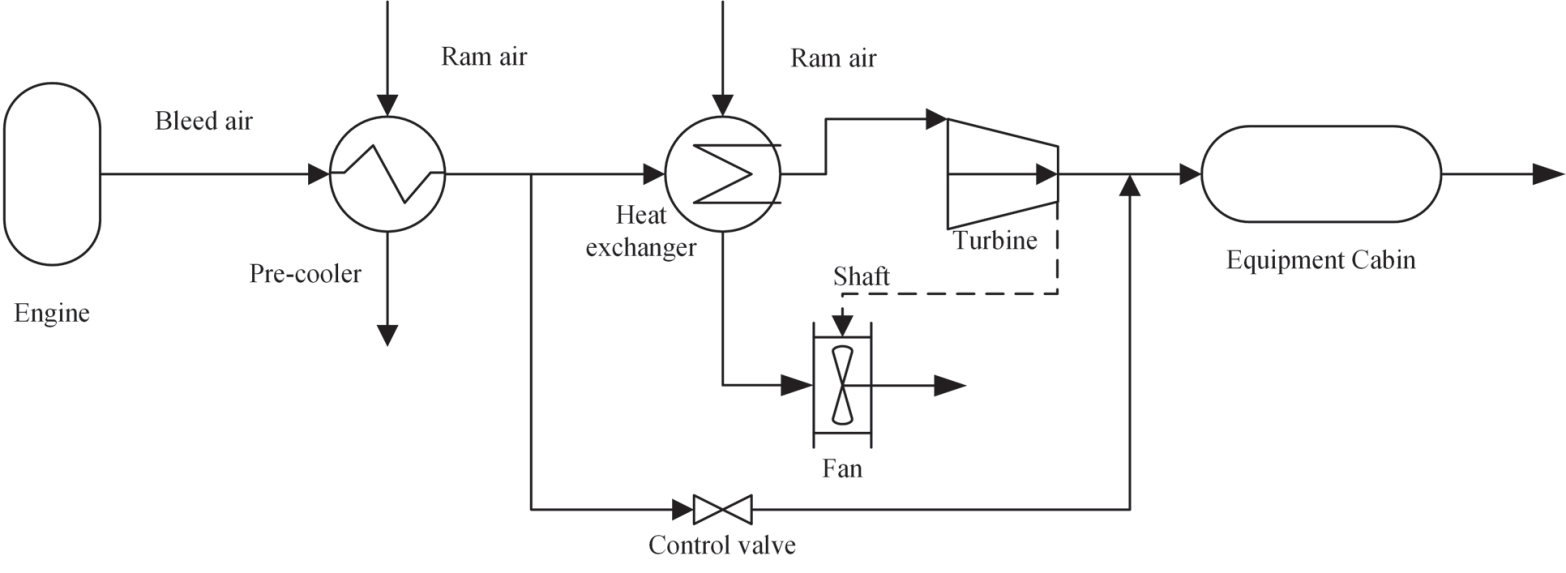
Structure of the chosen ECS.

Fig 1 shows the structure of the chosen ECS, which is a typical environmental control system for a fighter aircraft. Bleed air from the engine is regulated by refrigeration fluid in order to control the temperature inside the equipment cabin [1].

High temperature and high pressure air is bled from the compressor of the aircraft engine. After the preliminary cooling of the pre-cooler, the bleed air is divided into two parts: one conducted into the hot path ducts with a control valve, and the other into the cold path ducts with the heat exchanger and turbine. Air conducted into the cold path ducts is first cooled by allowing ram air to pass through the cold side of the heat exchanger, and is then cooled again during its expansion in the turbine, driving the fan to eject ram air through the shaft between them. The temperature of the air at the turbine outlet is extremely low. The control valve regulates the ratios of cold and hot air from the cold and hot paths, respectively, to the supply air with the appropriate temperatures for the refrigeration of the equipment cabin.

The air-to-air cross flow plate fin heat exchanger, the most commonly used in aviation, is chosen as the study case to demonstrate how the STF algorithm is realized in the ECS dynamic fault diagnosis.

**Fig 2:**
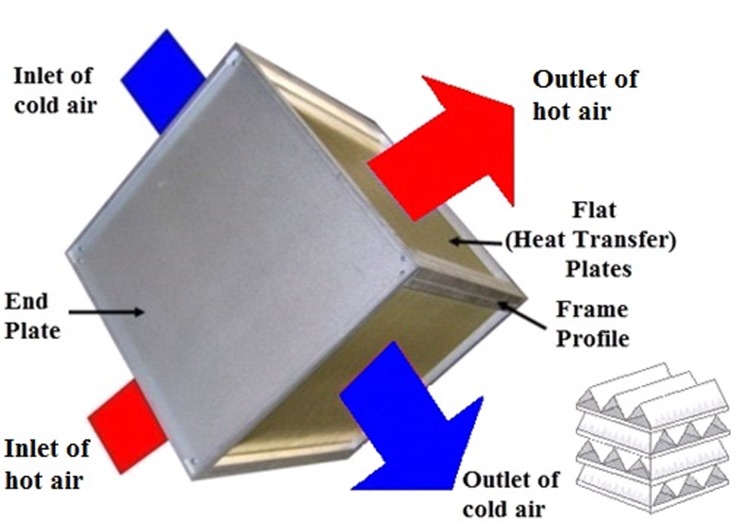
Structure of air-to-air cross flow plate fin heat exchanger.

In an air-to-air cross flow plate fin heat exchanger, the two airstreams (hot air and cold air) enter the heat exchanger perpendicular to one another and also travel perpendicular to one another through the heat exchanger, that is, the hot airstream flows through channels and the cold stream passes around the channels at a 90 º angle (see Fig 2).

### Nomenclature

*A*_*c*_, effective flow area of the cold air; *A*_*h*_, effective flow area of the hot air; *A*_*cw*_, effective heat exchange area of the cold side; *A*_*hw*_, effective heat exchange area of the hot side; *c*_*pc*_, specific heat of the cold air at constant pressure; *c*_*ph*_, specific heat of the hot air at constant pressure; *c*_*pw*_, specific heat of heat exchanger at constant pressure; *m*_*w*_, mass of heat exchanger; ${\dot{m}}_{c}$, mass flow rate of the cold air; ${\dot{m}}_{h}$, mass flow rate of the hot air; *t*_*bleed*_(*τ*), temperature of the engine bleed air; *t*_*ram*_(*τ*), temperature of the ram air from aircraft engine; *t*_*w*_(*τ*), surface temperature of heat exchanger; *t*_*c*.*in*_(*τ*), temperature of the cold air at the cold side inlet; *t*_*c*.*out*_(*τ*), temperature of the cold air at the cold side outlet; *t*_*pc*_(*τ*), average temperature of the cold air; *t*_*h*.*in*_(*τ*), temperature of hot air at the cold side inlet; *t*_*h*.*out*_(*τ*), temperature of hot air at the cold side outlet; *t*_*ph*_(*τ*), average temperature of the hot air; *v*_*c*_, velocity of the cold air; *v*_*h*_, velocity of the hot air.

### Greek Symbols

*α*_*c*_, convective heat transfer coefficient of the cold side; *α*_*h*_, convective heat transfer coefficient of the hot side; *γ*_1_, model parameter; *γ*_2_, model parameter; *γ*_3_, model parameter; *γ*_4_, model parameter; *η*_*c*_, heat transfer efficiency of the cold side surface; *η*_*h*_, heat transfer efficiency of the hot side surface; *ρ*_*c*_, cold air density; *ρ*_*h*_, hot air density; *τ*, time; Δ*τ*, time step.

### Subscripts

*c*, cold side or cold air; *h*, hot side or hot air; *w*, heat exchanger; *in*, at the inlet of the heat exchanger; *out*, at the outlet of the heat exchanger; *p*, average temperature of the cold or the hot air.

### Heat Exchanger Model

The heat exchanger model is based on the classical lumped parameter method [8]. The temperature of the cold air entering the heat exchanger is equal to the temperature of the ram air from the aircraft engine given as follows:

$$t_{c.in}\left(\tau \right)=t_{ram}\left(\tau \right).$$ (1)

As the cold air travels along the heat exchanger, its temperature at any point in the channel can be calculated by the following equation:

$${\dot{m}}_{c}c_{pc}dx\frac{\delta t_{c}\left(x,\tau \right)}{\delta x}+\eta_{c}\alpha_{c}A_{cw}\left[t_{c}\left(x,\tau \right)-t_{w}\right]=0.$$ (2)

After applying the Laplace transformation and inverse transformation to Eq. (2), the temperature of the cold air leaving the heat exchanger is obtained by Eq. (3) as follows:

$$t_{c.out}\left(\tau \right)=t_{w}\left(\tau \right)+\left[t_{c.in}\left(x,\tau \right)-t_{w}\left(\tau \right)\right]\cdot \text{exp}\left(-\frac{\gamma_{1}}{\gamma_{2}}\right),$$ (3)

where *γ*_1_ = *η*_*c*_ *α*_*c*_ *A*_*cw*_, $\gamma_{2}={\dot{m}}_{c}c_{pc}$, and ${\dot{m}}_{c}=\rho_{c}A_{c}v_{c}$.

After applying the Laplace transformation and inverse transformation to Eq. (2), the average temperature of the cold air *t*_*pc*_(*τ*) is given by the following:

$$t_{pc}\left(\tau \right)=t_{w}\left(\tau \right)+\left[t_{c.in}\left(\tau \right)-t_{w}\left(\tau \right)\right]\cdot \left[1-\exp\left(-\frac{\gamma_{1}}{\gamma_{2}}\right)\right]\cdot {\left(\frac{\gamma_{1}}{\gamma_{2}}\right)}^{-1}.$$ (4)

The hot air is bled from the compressor of the aircraft engine, so the temperature of the hot air entering the heat exchanger *t*_*h*.*in*_(*τ*) is equal to the temperature of the engine bleed air *t*_*bleed*_(*τ*) given as follows:

$$t_{h.in}\left(\tau \right)=t_{bleed}\left(\tau \right).$$ (5)

As the hot air travels along the heat exchanger, its temperature at any point in the channel can be calculated by the following equation:

$${\dot{m}}_{h}c_{ph}dx\frac{\delta t_{h}\left(x,\tau \right)}{\delta x}+\eta_{h}\alpha_{h}A_{hw}\left[t_{h}\left(x,\tau \right)-t_{w}\right]=0.$$ (6)

After applying the Laplace transformation and inverse transformation to Eq. (6), the temperature of the hot air leaving the heat exchanger is obtained by Eq. (7) as follows:

$$t_{h.out}\left(\tau \right)=t_{w}\left(\tau \right)+\left[t_{h.in}\left(x,\tau \right)-t_{w}\left(\tau \right)\right]\cdot \exp\left(-\frac{\gamma_{3}}{\gamma_{4}}\right),$$ (7)

where *γ*_3_ = *η*_*h*_ *α*_*h*_ *A*_*hw*_, $\gamma_{4}={\dot{m}}_{h}c_{ph}$, and ${\dot{m}}_{h}=\rho_{h}A_{h}v_{h}$.

After applying the Laplace transformation and inverse transformation to Eq. (6), the average temperature of the hot air is given by the following:

$$t_{ph}\left(\tau \right)=t_{w}\left(\tau \right)+\left[t_{h.in}\left(\tau \right)-t_{w}\left(\tau \right)\right]\cdot \left[1-\exp\left(-\frac{\gamma_{3}}{\gamma_{4}}\right)\right]\cdot {\left(\frac{\gamma_{3}}{\gamma_{4}}\right)}^{-1}.$$ (8)

The average temperature of the heat exchanger surface is obtained by Eq. (9) as follows:

$$m_{w}c_{pw}\frac{dt_{w}}{d\tau }=\eta_{c}\alpha_{c}A_{cw}\left(t_{pc}-t_{w}\right)+\eta_{h}\alpha_{h}A_{hw}\left(t_{ph}-t_{w}\right).$$ (9)

To facilitate calculation, Eq. (9) can be written as the following form of difference equation:

$$t_{w}^{\left(n\right)}=t_{w}^{\left(n-1\right)}+\frac{\gamma_{1}\Delta \tau }{m_{w}c_{pw}}\left(t_{pc}^{\left(n\right)}-t_{w}^{\left(n-1\right)}\right)+\frac{\gamma_{3}\Delta \tau }{m_{w}c_{pw}}\left(t_{ph}^{\left(n\right)}-t_{w}^{\left(n-1\right)}\right).$$ (10)

## Fault Information of Heat Exchanger

The basic idea behind fault detection and diagnosis is that numerous types of failures can be considered as changes in the coefficients of the process. These coefficients are implied by the model of the process parameters, and can be constant or time-varying. A system symptom analysis is required regardless of the method of fault diagnosis. Table 1 presents the details of the most common faults of the heat exchanger. Leakage and fouling of the heat exchanger have the same fault-related process parameters. For this reason, it is impossible for fault diagnosis algorithm to distinguish between leakage and fouling of the heat exchanger.

**Table 1:** Faults of heat exchanger.

Faults of the heat exchanger	Symptom of fault (fault-related coefficients of the process)	Diagnostic strategy (fault-related process parameters of the heat exchanger model)
**Leakage**	Sudden reduction of mass flow(${\dot{m}}_{c}$ or ${\dot{m}}_{h}$)	Real-time monitor changes of *ρ*_*h*_ *A*_*h*_ *v*_*h*_ *c*_*ph*_ (*γ*_*4*_) or *ρ*_*c*_ *A*_*c*_ *v*_*c*_ *c*_*pc*_(*γ*_*2*_)
**Blocking**	Decrease of heat transfer coefficient(*α*_*c*_ or *α*_*h*_)	Real-time monitor changes of *η*_*h*_ *α*_*h*_ *A*_*hw*_(*γ*_*3*_) or *η*_*c*_ *α*_*c*_ *A*_*cw*_(*γ*_*1*_)
**Fouling**	Significant change in value of effective flow area (*A*_*h*_ or *A*_*c*_)	Real-time monitor changes of *ρ*_*h*_ *A*_*h*_ *v*_*h*_ *c*_*ph*_(*γ*_*4*_) or *ρ*_*c*_ *A*_*c*_ *v*_*c*_ *c*_*pc*_(*γ*_*2*_)

## Model Modification and State Estimation

The model modification and state estimation method to be presented here is detailed by Pang et.al. [8]. It is modified to employ the DMF algorithm in the heat exchanger diagnosis. This model can also be used by the STF in the heat exchanger diagnosis. To make the present paper self-contained, a brief review of this method is needed.

The system observation equation has the following form:

$$\left[\begin{array}{c} t_{c.out}\left(k\right)\\ t_{h.out}\left(k\right) \end{array}\right]=\left[\begin{array}{c} t_{w}\left(k\right)+\left[t_{c.in}\left(k\right)-t_{w}\left(k\right)\right]\cdot \exp\left(-\frac{\gamma_{1}\left(k\right)}{\gamma_{2}\left(k\right)}\right)\\ t_{w}\left(k\right)+\left[t_{h.in}\left(k\right)-t_{w}\left(k\right)\right]\cdot \exp\left(-\frac{\gamma_{3}\left(k\right)}{\gamma_{4}\left(k\right)}\right) \end{array}\right]+\left[\begin{array}{c} n_{tc.out}\left(k\right)\\ n_{th.out}\left(k\right) \end{array}\right],$$ (11)

where *n*_*tc*.*out*_(*k*) and *n*_*th*.*out*_(*k*) represent the noises of *t*_*c*.*out*_(*k*) and *t*_*h*.*out*_(*k*) respectively, which are assumed for uncorrelated, zero-mean, Gaussian white noise.

The state equations of the heat exchanger have the following forms:

$$\left[\begin{array}{c} \gamma_{1}\left(k\right)\\ \gamma_{2}\left(k\right)\\ \gamma_{3}\left(k\right)\\ \gamma_{4}\left(k\right)\\ t_{pc}\left(k\right)\\ t_{ph}\left(k\right)\\ t_{w}\left(k\right) \end{array}\right]=\left[\begin{array}{c} \gamma_{1}\left(k-1\right)\\ \gamma_{2}\left(k-1\right)\\ \gamma_{3}\left(k-1\right)\\ \gamma_{4}\left(k-1\right)\\ f_{1}\left(X\left(k-1\right)\right)\\ f_{2}\left(X\left(k-1\right)\right)\\ f_{3}\left(X\left(k-1\right)\right) \end{array}\right]+\left[\begin{array}{c} n_{\gamma_{1}}\left(k-1\right)\\ n_{\gamma_{2}}\left(k-1\right)\\ n_{\gamma_{3}}\left(k-1\right)\\ n_{\gamma_{4}}\left(k-1\right)\\ 0\\ 0\\ n_{t_{w}}\left(k-1\right) \end{array}\right],$$ (12)

To facilitate calculation, Eq. (4) can be written as the following form of difference equation:

$$f_{1}\left(X\left(k-1\right)\right)=t_{w}\left(k-1\right)+\left[t_{c.in}\left(k\right)-t_{w}\left(k-1\right)\right]\cdot \left[1-\exp\left(-\frac{\gamma_{1}\left(k-1\right)}{\gamma_{2}\left(k-1\right)}\right)\right]\cdot {\left[\frac{\gamma_{1}(k-1)}{\gamma_{2}(k-1)}\right]}^{-1}.$$ (13)

To facilitate calculation, Eq. (8) can be written as follows:

$$f_{2}\left(X\left(k-1\right)\right)=t_{w}\left(k-1\right)+\left[t_{h.in}\left(k\right)-t_{w}\left(k-1\right)\right]\cdot \left[1-\exp\left(-\frac{\gamma_{3}\left(k-1\right)}{\gamma_{4}\left(k-1\right)}\right)\right]\cdot {\left[\frac{\gamma_{3}(k-1)}{\gamma_{4}(k-1)}\right]}^{-1}.$$ (14)

Eq. (10) can be written as the following form:

$$f_{3}\left(X\left(k-1\right)\right)=t_{w}\left(k-1\right)+\frac{\gamma_{1}\left(k-1\right)T}{m_{w}c_{pw}}\cdot \left[t_{pc}\left(k-1\right)-t_{w}\left(k-1\right)\right]+\frac{\gamma_{3}\left(k-1\right)T}{m_{w}c_{pw}}\cdot \left[t_{ph}\left(k-1\right)-t_{w}\left(k-1\right)\right].$$ (15)

$$X\left(k\right)={\left[\begin{array}{ccccccc} \gamma_{1}\left(k\right) & \gamma_{2}\left(k\right) & \gamma_{3}\left(k\right) & \gamma_{4}\left(k\right) & t_{pc}\left(k\right) & t_{ph}\left(k\right) & t_{w}\left(k\right) \end{array}\right]}^{T}.$$ (16)

$n_{\gamma_{1}}$, $n_{\gamma_{2}}$, $n_{\gamma_{3}}$, $n_{\gamma_{4}}$ and $n_{t_{w}}$, respectively represent uncorrelated, zero-mean Gaussian white noises; and T is the sampling interval.

## Fault Detection and Diagnosis Strategy

In Eq. (12), the heat exchanger related parameters *γ*_*1*_, *γ*_*2*_, *γ*_*3*,_ and *γ*_*4*_ are unknown. Based on the STF (see S1 Appendix) proposed in [10], we can do an on-line estimation of the parameters *γ*_*1*_, *γ*_*2*_, *γ*_*3*,_ and *γ*_*4*_ as follows:

$$\hat{x}\left(k+1|k+1\right)=\hat{x}\left(k+1|k\right)+K\left(k+1\right)\upsilon \left(k+1\right),$$ (17)

where *K*(*k* + 1) is the filter gain calculated online by STF, *υ*(*k* + 1) represents the residual error.

Based on the MB classification algorithm (see S2 Appendix) [10], a fault detection and diagnosis algorithm is provided, which can be used to detect, isolate, and estimate the heat exchanger faults.

*Step 1 (fault-related parameter estimation)* From Eq. (17), we obtain the following heat exchanger fault parameter estimator:

$$\begin{array}{l} \hat{x}\left(k+1|k+1\right)={\left[\begin{array}{ccccccc} {\hat{x}}_{1}\left(k\right) & {\hat{x}}_{2}\left(k\right) & {\hat{x}}_{3}\left(k\right) & {\hat{x}}_{4}\left(k\right) & {\hat{x}}_{5}\left(k\right) & {\hat{x}}_{6}\left(k\right) & {\hat{x}}_{7}\left(k\right) \end{array}\right]}^{T}\\ ={\left[\begin{array}{ccccccc} {\hat{\gamma }}_{1}\left(k\right) & {\hat{\gamma }}_{2}\left(k\right) & {\hat{\gamma }}_{3}\left(k\right) & {\hat{\gamma }}_{4}\left(k\right) & {\hat{t}}_{pc}\left(k\right) & {\hat{t}}_{ph}\left(k\right) & {\hat{t}}_{w}\left(k\right) \end{array}\right]}^{T} \end{array}.$$ (18)

*Step 2 (fault detection and isolation)* Based on ${\hat{x}}_{i}\left(k\right)$, *i* = 1,2,3,4, we conduct the following tests for *γ*_*1*_, *γ*_*2*_, *γ*_*3*,_ and *γ*_*4*_:

- If *H*_*1*_ (MB algorithm) of *γ*_*i*_ alarms, the fault-related parameter *γ*_*i*_ happens, and we proceed to Step 3;
- If the aforementioned condition is not satisfied, we return to Step 1.

*Step 3 (fault amplitude estimation)* The equivalent heat exchanger fault-related parameter *γ*_*i*_ is estimated to be the estimated equivalent fault amplitude (EEFA) as follows:

$$\text{EEFA}:\;{\hat{\gamma }}_{i}\left(k\right)-\gamma_{i}^{0},$$ (19)

after which we return to Step 1. $\gamma_{i}^{0}$ represents the value of *γ*_*i*_ when heat exchanger is under normal state.

S3 Appendix shows details of the fault detection and diagnosis strategy.

## Results and Discussion

In this section, the effectiveness of the proposed fault diagnosis method is thoroughly studied through simulated data from the cross-flow heat exchanger in case the flight altitude is 2 km.

The model parameters of the heat exchanger are reported in Table 2.

**Table 2:** Model parameters of the heat exchanger.

Model parameter	The hot side	The cold side
**Plate distance (mm)**	5	7.5
**Number of fin layers**	7	8
**Fin thickness (mm)**	0.15	0.15
**Plate thickness (mm)**	0.5	0.5
**Side bar thickness (mm)**	2	2
**Length of channel (mm)**	260	100
**Inlet temperature (ºC)**	250	90
**Mass flow (kg/s)**	0.1778	1
**Initial surface temperature of heat exchanger (ºC)**	140	140

The initial values of the STF are selected as described below.

Measurement noise (*n*_*tc*.*out*_,*n*_*th*.*out*_) and system noise ($n_{\gamma_{1}}$, $n_{\gamma_{2}}$, $n_{\gamma_{3}}$, $n_{\gamma_{4}}$, and $n_{t_{w}}$) are types of Gaussian white noise with the following statistics:

$$\begin{array}{l} n_{tc.out}∼N(0,1),\;n_{th.out}∼N(0,1),\;n_{\gamma_{1}}∼N(0,0.01),\;n_{\gamma_{2}}∼N(0,0.01),\;n_{\gamma_{3}}∼N(0,0.01),\\ n_{\gamma_{4}}∼N(0,0.01),\;n_{t_{w}}∼N(0,0.01). \end{array}$$

The parameters of the MB algorithm are selected as follows:

$$\begin{array}{l} \gamma_{1}{}^{0}=577.2,\;\gamma_{2}{}^{0}=1008.0,\;\gamma_{3}{}^{0}=677.0,\;\gamma_{4}{}^{0}=183.7,\;\sigma_{\gamma_{1}{}^{0}}^{2}=\sigma_{\gamma_{2}{}^{0}}^{2}=\sigma_{\gamma_{3}{}^{0}}^{2}=\sigma_{\gamma_{4}{}^{0}}^{2}=0.01,\\ N_{1}=10;\;\beta_{\gamma_{1}}=\beta_{\gamma_{2}}=\beta_{\gamma_{3}}=\beta_{\gamma_{4}}=10000. \end{array}$$

The results of the proposed methods when the heat exchanger runs normally are shown in S1 and S2 Figs. It is illustrated that the initial values of the STF algorithm and the parameters of the MB algorithm have been selected well.

**Table 3:** Faults to be tested.

Faults	Description
***F***_***1***_	Blocking of the cold side of the heat exchanger. At *k* = 400, *γ*_*1*_ jump from 577.2 to519.5 (0.9*577.2)
***F***_***2***_	Leakage or fouling of the cold side of the heat exchanger. At *k* = 400, *γ*_*2*_ jump from 1008.0 to705.6 (0.7*1008.0)
***F***_***3***_	Blocking of the hot side of the heat exchanger. At *k* = 400, *γ*_*3*_ jump from 677.0 to473.9 (0.7*677.0)
***F***_***4***_	Leakage or fouling of the hot side of the heat exchanger. At *k* = 400, *γ*_*4*_ jump from 183.7 to128.6 (0.7*183.7)
***F***_***5***_	Blocking of the cold side of the heat exchanger. From *k* = 400 to 600, *γ*_*1*_ decrease 0.2885 at each steps from 577.2 to519.5 (0.9*577.2)
***F***_***6***_	Leakage or fouling of the cold side of the heat exchanger. From *k* = 400 to 600, *γ*_*2*_ decrease1.5120 at each steps from 1008.0 to705.6 (0.7*1008.0)
***F***_***7***_	Blocking of the hot side of the heat exchanger. From *k* = 400 to 600, *γ*_*3*_ decrease1.0155 at each steps from 677.0 to473.9 (0.7*677.0)
***F***_***8***_	Leakage or fouling of the hot side of the heat exchanger. From *k* = 400 to 600, *γ*_*4*_ decrease 0.2755 at each steps from183.7 to128.6 (0.7*183.7)
***F***_***9***_	Blocking of the cold side of the heat exchanger. At *k* = 400, *γ*_*1*_ jump from 577.2 to461.8(0.8*577.2)

Nine kinds of heat exchanger faults were tested. The faults are summarized in Table 3. The first four faults are abrupt faults, and “*F*_*5*_” to “*F*_*8*_” are gradual faults. “*F*_*9*_”is more serious than “*F*_*1*_” and is used to test the efficiency of the proposed algorithm in order to identify different severity faults. The simulation results are shown in Table 4. All of the faults were detected, isolated, and estimated without false alarm and missing alarm.

**Table 4:** Simulation results.

Faults	F_*d*_ (k =)	F_*e*_ (k =)	EEFA		
			Value (real)	Value (estimated)	Accuracy
***F***_***1***_	415	461	-57.7	-56.2	97.400%
***F***_***2***_	404	457	-302.4	-302.4	99.636%
***F***_***3***_	412	465	-203.1	-203.1	99.705%
***F***_***4***_	412	482	-54.9	-54.9	99.637%
***F***_***5***_	489	684	-57.7	-54.2	93.934%
***F***_***6***_	489	701	-302.4	-298	98.545%
***F***_***7***_	486	690	-203.1	-198.7	97.834%
***F***_***8***_	489	657	-54.9	-55.0	99.818%
***F***_***9***_	410	469	-115.4	-114.2	98.960%

*F*_*d*_: fault detected time; *F*_*e*_: fault estimated time; EEFA: estimated equivalent fault amplitude.

The simulation results of the proposed methods under faults “*F*_*1*_” and“*F*_*8*_” are shown in S3–S6 Figs, respectively. S7 Fig indicates that the STF can successfully distinguish “*F*_*1*_” (S7A Fig) and “*F*_*9*_” (S7B Fig). For comparison, S8B Fig presents the simulation results of fault diagnosis based on the DMF under the fault “*F*_*1*_,” which verifies that the proposed method performs better than the DMF.

From the deductions derived from the STF, MB, normalization methods and computer simulations, we have obtained the following findings.

1) The jump type fault of the heat exchanger can be detected extremely fast; it typically requires approximately 10 steps to detect the fault after it occurs (see Table 4). For fault estimation, a significant amount of time is needed to obtain accurate fault amplitude. In this example, the estimation accuracy of the fault amplitude has exceeded 93% in all cases (see Table 4).

2) The incipient fault can also be detected at an early stage. For example, fault *F*_*8*_ is detected at *k* = 489. At this time, the real fault amplitude is only 159.2 ≈0.867*183.7. It is possible to detect a smaller fault if we select a lower threshold $\beta_{\gamma_{i}}$, but the rate of the false alarm is expected to increase.

3) The design procedure of the heat exchanger fault diagnosis strategy is simple, consisting only of two parts: a) using the combined estimation algorithm of parameter and state (STF) to estimate the states and fault-related parameters of the heat exchanger online, and b) using the heat exchanger parameter estimation result to form the fault detection and diagnosis algorithm (MB).

## Conclusions

A heat exchanger fault diagnosis approach based on STF and MB was proposed in this study. A modified parameter estimation method based on STF is utilized to adaptively estimate the heat exchanger fault parameters. To further realize the fault detection and failure mode classification, an MB algorithm is developed. The results demonstrate that the proposed methods can be used to diagnose both the slow drift and abrupt heat exchanger faults, identify different fault severities, and obtain accurate fault amplitude.

This approach are capable of providing accurate, stable, and rapid fault diagnosis of the heat exchanger and can reveal online whether the heat exchanger is in a faulty state. Such information is valuable in improving the maintainability of the heat exchanger.

In the future, the authors plan to apply the proposed method to other types of heat exchanger in the ECS and evaluate the possibility of generalizing the proposed method, in order to further improve the reliability of the ECS. Furthermore, the authors also expect to verify the effectiveness and applicability using practical engineering data of the heat exchanger for improving the maturity of the proposed method.

## Supporting Information

S1 Fig Estimation of tc.out, th.out, γ1, γ2, γ3, and γ4: no faults, STF is used. (TIF)

S2 Fig Fault detection and diagnosis results: no faults, MB is used. (TIF)

S3 Fig Estimation of *t*_*c*.*out*_, *t*_*h*.*out*_, *γ*_*1*_, *γ*_*2*_, *γ*_*3*,_ and *γ*_*4*_: fault *F*_*1*_ occurs, STF is used. (TIF)

S4 Fig Fault detection and diagnosis results: fault *F*_*1*_ occurs, MB is used. (TIF)

S5 Fig Estimation of *t*_*c*.*out*_, *t*_*h*.*out*_, *γ*_*1*_, *γ*_*2*_, *γ*_*3*,_ and *γ*_*4*_: fault *F*_*8*_ occurs, STF is used. (TIF)

S6 Fig Fault detection and diagnosis results: fault *F*_*8*_ occurs, MB is used. (TIF)

S7 Fig Estimation of *γ*_*1*_, fault detection and diagnosis results: fault *F*_*9*_ (a) and fault *F*_*1*_ (b) occur. (TIF)

S8 Fig Estimation of *γ*_*1*_, fault detection and diagnosis results: fault *F*_*9*_ occurs, STF (a) and DMF (b) are used. (TIF)

S9 Fig Details of the Fault Detection and Diagnosis Strategy. (TIF)

S1 Appendix Strong Tracking Filter (STF)-Based State and Parameter Estimation. (DOC)

S2 Appendix MB Classification Algorithm. (DOC)

S3 Appendix Details of the Fault Detection and Diagnosis Strategy. (DOC)
