# Supplementary figures and images for: Fault Diagnosis for the Heat Exchanger of the Aircraft Environmental Control System Based on the Strong Tracking Filter
Source: PLoS One. 2015 Mar 30;10(3):e0122829. doi: 10.1371/journal.pone.0122829 (PMC4379147; doi:10.1371/journal.pone.0122829)

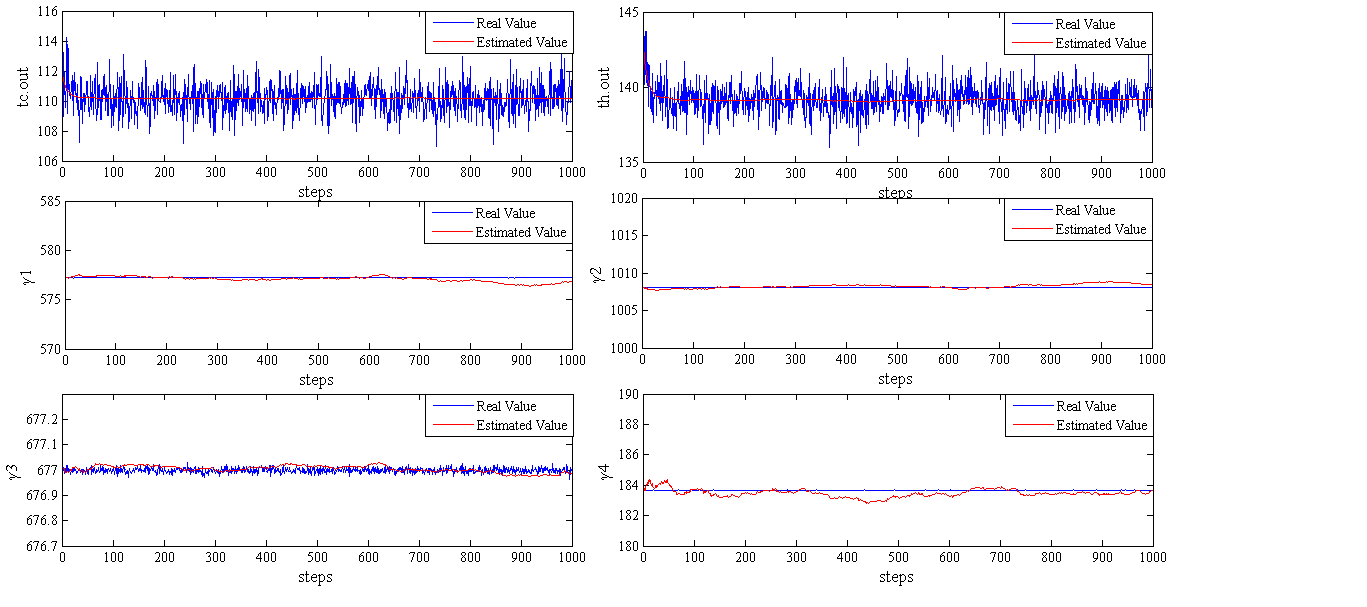

Supplement: S1 Fig — (TIF) [file pone.0122829.s001.tif]

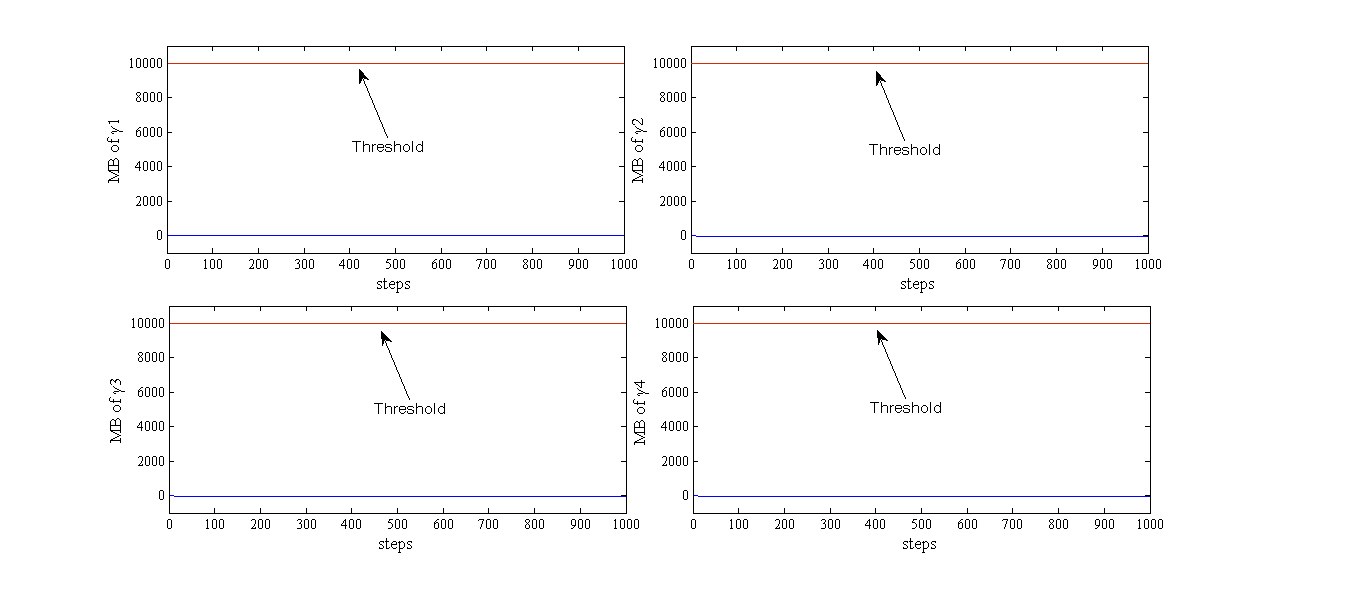

Supplement: S2 Fig — (TIF) [file pone.0122829.s002.tif]

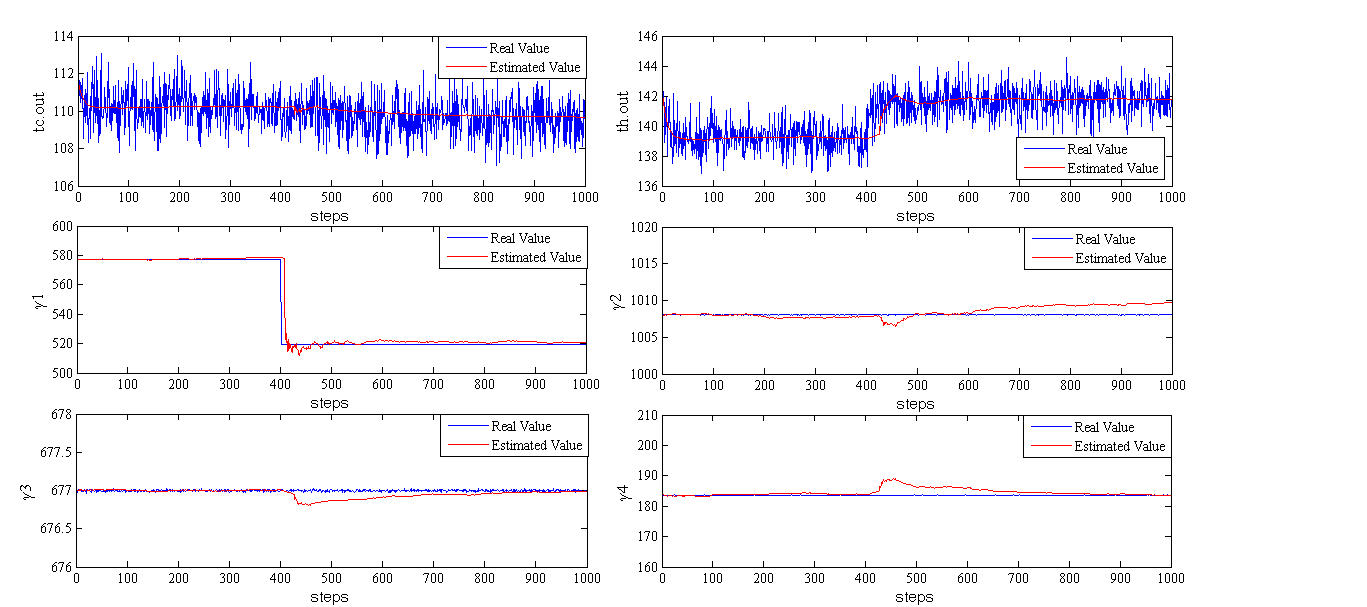

Supplement: S3 Fig — (TIF) [file pone.0122829.s003.tif]

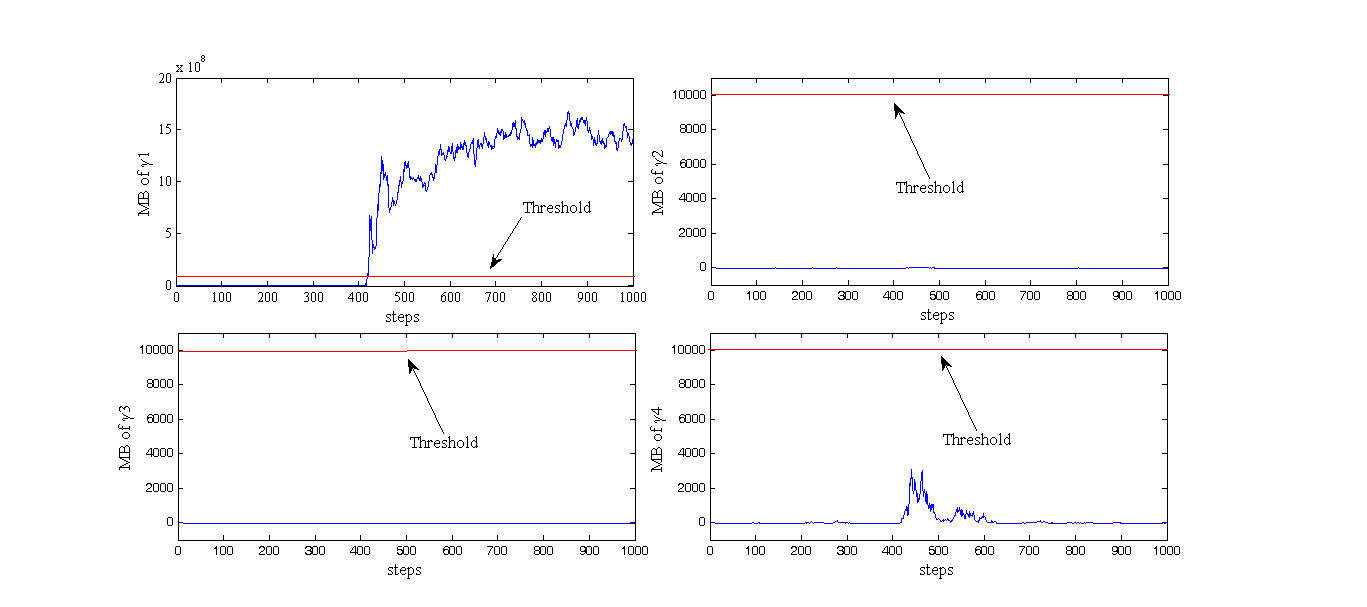

Supplement: S4 Fig — (TIF) [file pone.0122829.s004.tif]

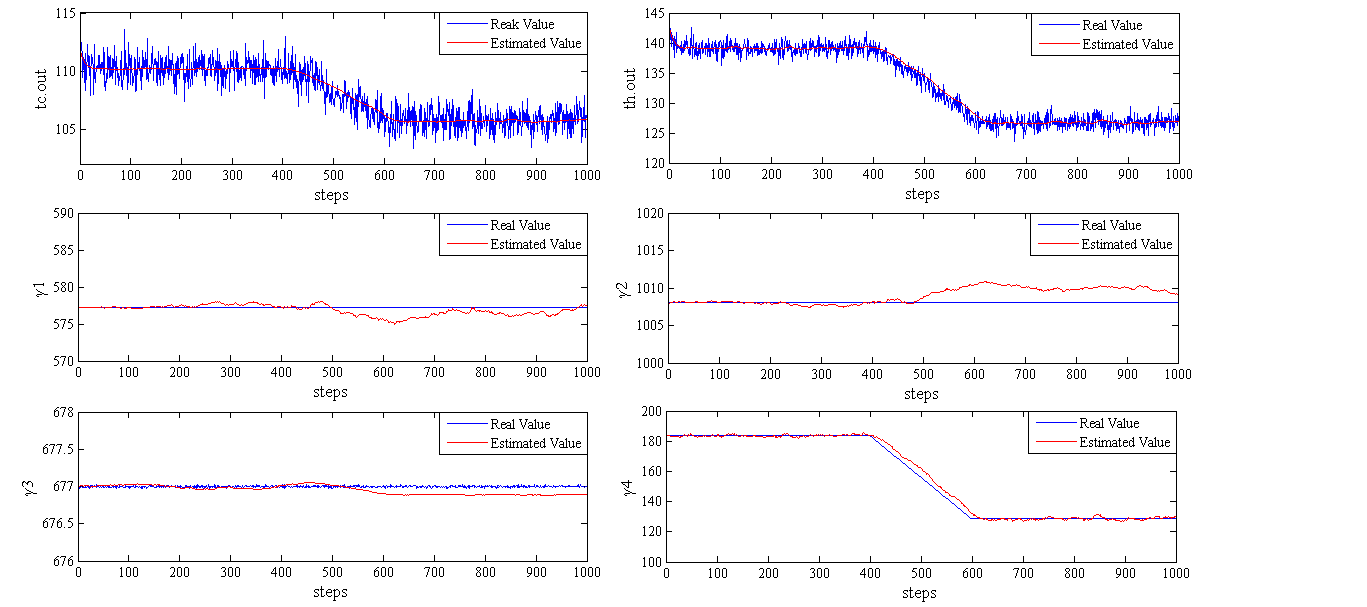

Supplement: S5 Fig — (TIF) [file pone.0122829.s005.tif]

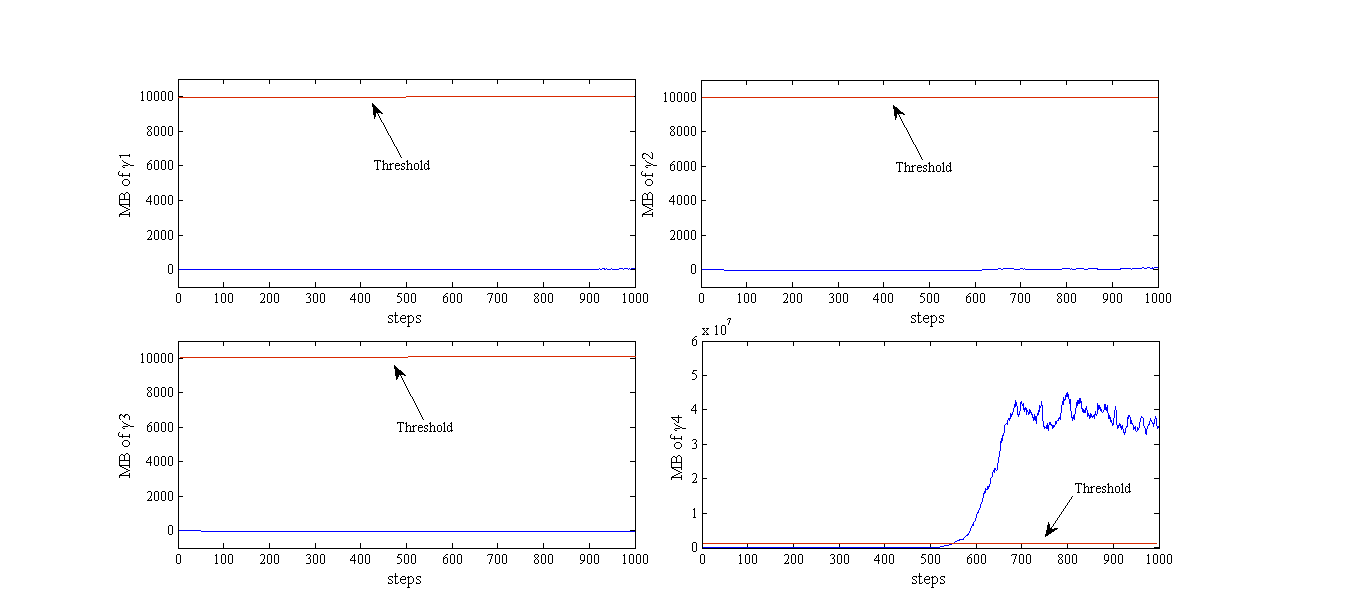

Supplement: S6 Fig — (TIF) [file pone.0122829.s006.tif]

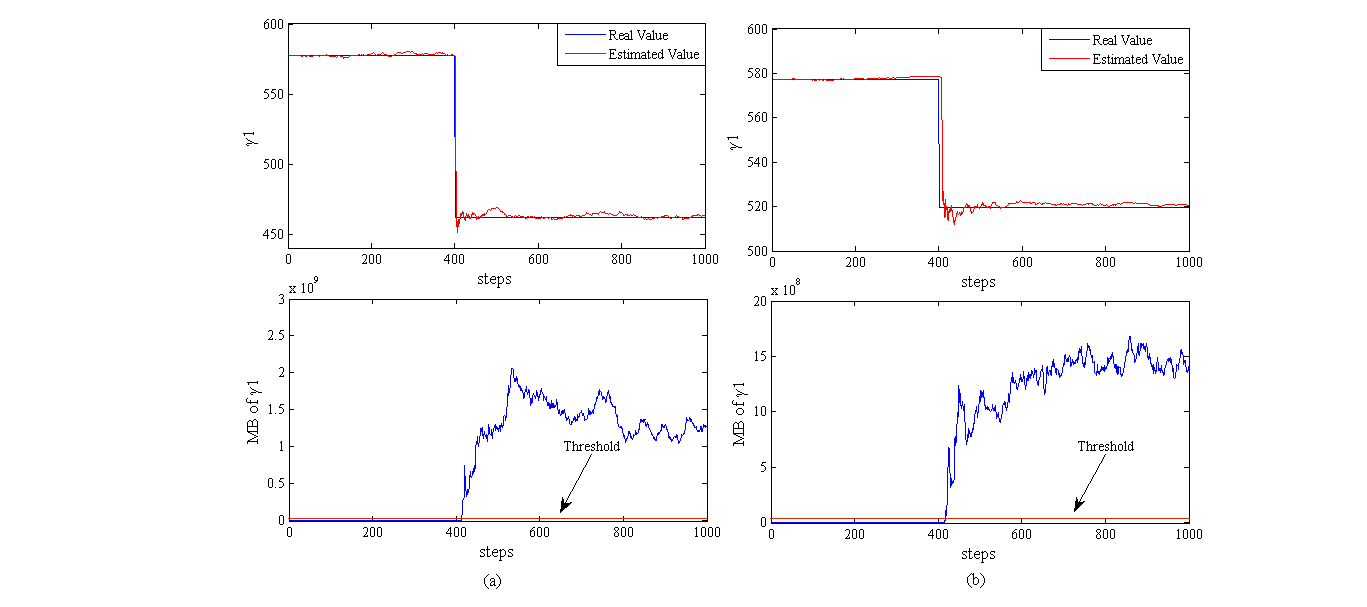

Supplement: S7 Fig — (TIF) [file pone.0122829.s007.tif]

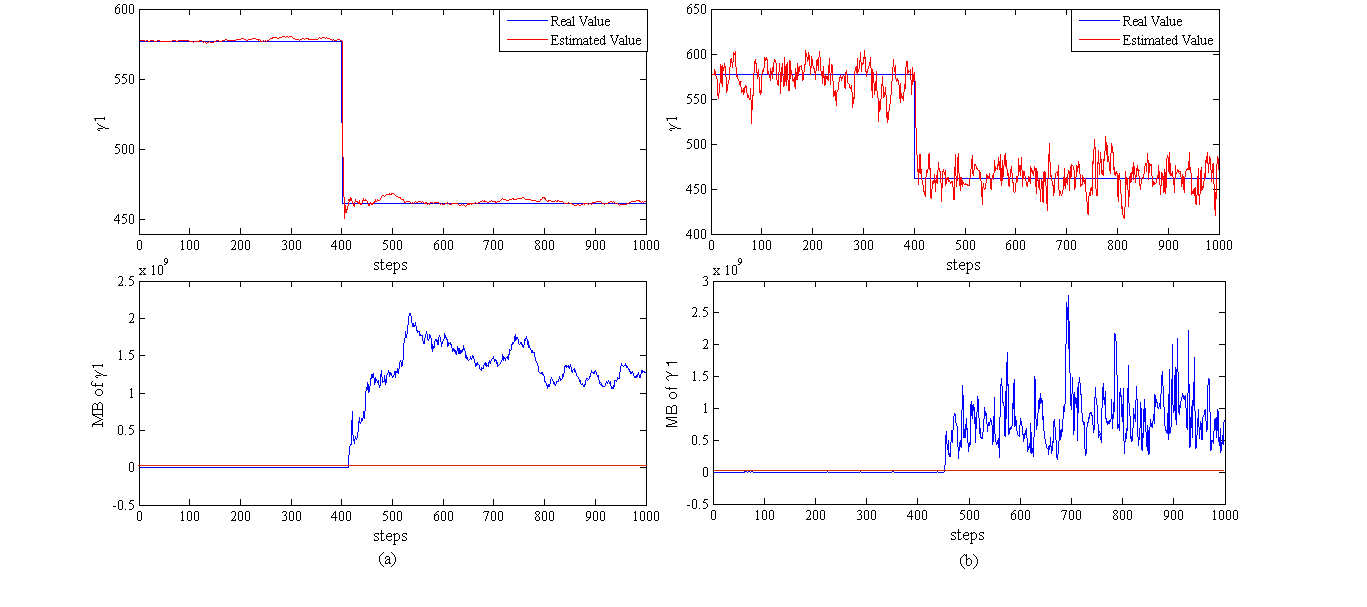

Supplement: S8 Fig — (TIF) [file pone.0122829.s008.tif]

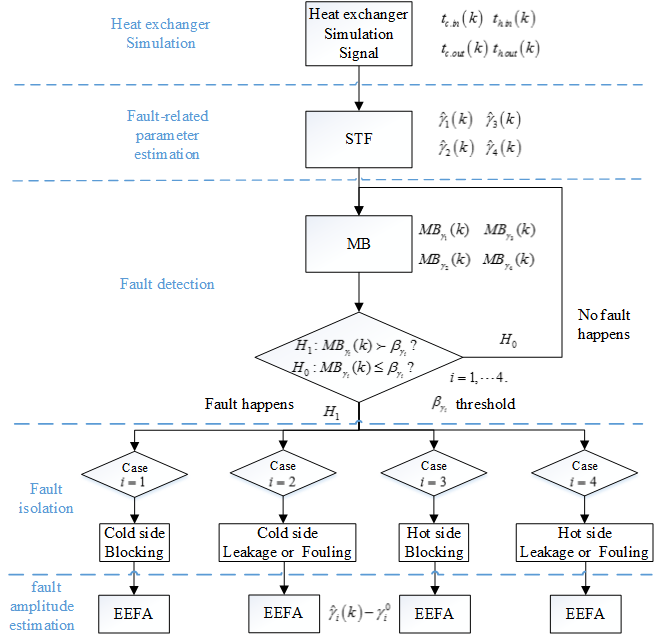

Supplement: S9 Fig — (TIF) [file pone.0122829.s009.tif]
