# Supplementary material for: Fault Diagnosis for the Heat Exchanger of the Aircraft Environmental Control System Based on the Strong Tracking Filter
Source: PLoS One. 2015 Mar 30;10(3):e0122829. doi: 10.1371/journal.pone.0122829 (PMC4379147; doi:10.1371/journal.pone.0122829)
Supplement: S1 Appendix — (DOC) [file pone.0122829.s010.doc]

**Appendix A. Strong Tracking Filter (STF)-Based State and Parameter Estimation**

Consider a class of nonlinear time-varying stochastic systems that can be described by the following discrete state space models:

$x\left(k+1\right)=f\left(k,u\left(k\right),x\left(k\right)\right)+\Gamma\left(k\right)v\left(k\right)$, (20)

$y\left(k+1\right)=g\left(k+1,x\left(k+1\right)\right)+e\left(k+1\right)$, (21)

where state$x\in\text{IR}^{n}$, input$u\in\text{IR}^{p}$, output$y\in\text{IR}^{m}$, nonlinear functions$f:\text{IR}^{n}\times\text{IR}^{p}\to\text{IR}^{n}$,$g:\text{IR}^{n}\to\text{IR}^{m}$, the process noise$v\in\text{IR}^{q}$ is a zero-mean, Gaussian white noise with covariance *Q*, the measurement noise$e\in\text{IR}^{m}$ is also a zero-mean, Gaussian white noise with covariance *R*, *Г*is a known matrix with proper dimension, and *v* and *e* are statistically independent variables.

A class of STFs for the nonlinear systems Eq. (20) and Eq. (21) should have the following general structure:

$\hat{x}\left(k+1\left|k+1\right.\right)=\hat{x}\left(k+1\left|k\right.\right)+K\left(k+1\right)\upsilon\left(k+1\right)$, (22)

where

$\hat{x}\left(k+1\left|k\right.\right)=f\left(k,u\left(k\right),\hat{x}\left(k\left|k\right.\right)\right)$, (23)

$\upsilon\left(k+1\right)=y\left(k+1\right)-g\left(k+1,\hat{x}\left(k+1\left|k\right.\right)\right)$, (24)

where *K(k+1)* denotes the gain matrix of STF, and *υ(k+1)* is the residual error vector. The problem is determining the time-varying gain matrix *K(k+1)* online to turn the filter into an STF.

A sufficient condition to make the filter Eq. (22) an STF is that the online determined *K(k+1)* should satisfy the following conditions:

, (25)

, (26)

where condition Eq. (26) indicates that the residual error vectors taken at each step should be made orthogonal to each other. The orthogonality principle has a profound physical meaning, that is, when the model/plant mismatch is large, the *K(k+1)* should be adjusted online, wherein the residual error vector has the statistics, such as white noise [i.e., Eq. (26) is satisfied], indicating that all useful information has been extracted from residual error vectors. It has been proven that if estimation is based on an accurate system model and only the common recursive least square (RLS) or Kalman filter approach is used, then Eq. (26) is naturally satisfied.

Based on the orthogonality principle and through deduction, an approximate algorithm for determining *K(k+1)* is obtained as follows:

$K\left(k+1\right)=\frac{P\left(k+1\left|k\right.\right)G^{T}\left(k+1,\hat{x}\left(k+1\left|k\right.\right)\right)}{G\left(k+1,\hat{x}\left(k+1\left|k\right.\right)\right)P\left(k+1\left|k\right.\right)G^{T}\left(k+1,\hat{x}\left(k+1\left|k\right.\right)\right)+R\left(k\right)}$, (27)

where

$P\left(k+1\left|k\right.\right)=L\left(k+1\right)F\left(k,u\left(k\right),\hat{x}\left(k\left|k\right.\right)\right)P\left(k\left|k\right.\right)F^{T}\left(k,u\left(k\right),\hat{x}\left(k\left|k\right.\right)\right)+\Gamma\left(k\right)Q\left(k\right)\Gamma^{T}\left(k\right)$, (28)

$P\left(k+1\left|k+1\right.\right)=\left[I_{n}-K\left(k+1\right)G\left(k+1,\hat{x}\left(k+1\left|k\right.\right)\right)\right]P\left(k+1\left|k\right.\right)$, (29)

$F\left(k,u\left(k\right),\hat{x}\left(k\left|k\right.\right)\right)=\frac{\delta f\left(k,u\left(k\right),x\left(k\right)\right)}{\delta x}\left|_{x=\hat{x}\left(k\left|k\right.\right)}\right.$, (30)

$G\left(k+1,\hat{x}\left(k+1\left|k\right.\right)\right)=\frac{\delta g\left(k+1,x\left(k+1\right)\right)}{\delta x}\left|_{x=\hat{x}\left(k+1\left|k\right.\right)}\right.$, (31)

$L\left(k+1\right)=diag\left\{\lambda_{1}\left(k+1\right),\lambda_{2}\left(k+1\right),\ldots,\lambda_{n}\left(k+1\right)\right\}$, (32)

$\begin{matrix} \lambda_{1}=\left\{\begin{matrix} \zeta_{i}\eta\left(k+1\right); & \zeta_{i}\eta\left(k+1\right)>1 \\ 1; & \zeta_{i}\eta\left(k+1\right)\leq1 \end{matrix}\right. \\ \begin{matrix} \begin{matrix} \begin{matrix} & \end{matrix} & \end{matrix} & i=1,2,\cdots,n \end{matrix} \end{matrix}$, (33)

$\eta\left(k+1\right)=\frac{tr\left[N\left(k+1\right)\right]}{\sum_{i=1}^{n} \zeta_{i}d_{ii}\left(k+1\right)}$, (34)

$N\left(k+1\right)=V_{0}\left(k+1\right)-\beta R\left(k+1\right)-G\left(k+1,\hat{x}\left(k+1\left|k\right.\right)\right)\Gamma\left(k\right)Q\left(k\right)\Gamma^{T}\left(k\right)G^{T}\left(k+1,\hat{x}\left(k+1\left|k\right.\right)\right)$, (35)

(36)

$V_{0}\left(k+1\right)=E\left[\upsilon\left(k+1\right)\upsilon^{T}\left(k+1\right)\right]$, (37)

$\approx\left\{\begin{matrix} \upsilon\left(1\right)\upsilon^{T}\left(1\right); & k=0 \\ \left(\rho V_{0}\left(k\right)+\upsilon\left(k+1\right)\upsilon^{T}\left(k+1\right)\right)/\left(1+\rho\right); & k\geq1 \end{matrix}\right.$, (38)

where *I_n_* is a ($n\times n$) unit matrix, $\rho=0.95$ is a forgetting factor, and $\zeta_{i}\geq1,i=1,2,\cdots n,$are predetermined coefficients. These coefficients can be roughly determined using a priori knowledge of the process. If we know that a state *x_j_* changes faster than others, a larger coefficient *ζ_i_* corresponding to this state should be selected, indicating that a priori knowledge of the process can be used in the STF. In case there is no a priori knowledge of the process, all the coefficients can be selected as “1”. The STF might be considered as a closed-loop filter, because the filter gain *K(k+1)* is adaptively calculated online according to the orthogonality principle. This is the main reason why the STF has strong robustness against model/plant parameter mismatches. By contrast, the Kalman Filter is an open-loop filter, because the gain matrix of the KF can be calculated offline only according to *(A,B,C)* of the dynamic processes, stored in computers, and then used later online.
