# Supplementary material for: Fault Diagnosis for the Heat Exchanger of the Aircraft Environmental Control System Based on the Strong Tracking Filter
Source: PLoS One. 2015 Mar 30;10(3):e0122829. doi: 10.1371/journal.pone.0122829 (PMC4379147; doi:10.1371/journal.pone.0122829)
Supplement: S2 Appendix — (DOC) [file pone.0122829.s011.doc]

**Appendix B. MB Classification Algorithm**

Based on the MB classification algorithm, a fault diagnosis and detection algorithm is provided, which can be used to detect, isolate, and estimate the heat exchanger fault.

We assume that the estimated system parameters at normal states follow normal distribution as follows:

$\hat{\gamma}(k|k)=\left[\begin{matrix} \hat{\gamma}_{1}(k|k) \\ \hat{\gamma}_{2}(k|k) \\ \vdots\\ \hat{\gamma}_{4}(k|k) \end{matrix}\right],\hat{\gamma}_{i}(k|k)\sim N(\gamma_{i}^{0},\sigma_{\gamma_{i}^{0}}^{2}),\mathrm{i}=1,\cdots,4.$, (39)

We define $MB_{\gamma_{i}}(k)$ as fault indicator for each of the estimated system parameters, *i*=1,2,3,4 as follows:

$MB_{\gamma_{i}}(k)=\frac{\sigma_{\gamma_{i}1}^{2}(k)}{\sigma_{\gamma_{i}^{0}}^{2}}-\ln\frac{\sigma_{\gamma_{i}2}^{2}(k)}{\sigma_{\gamma_{i}^{0}}^{2}}-1$, (40)

where

$\sigma_{\gamma_{i}1}^{2}(k)=\frac{1}{N_{1}-1}\sum_{j=1}^{N_{1}} \left[\hat{\gamma}_{i}(k-j|k-j)-\gamma_{i}^{0}\right]^{2}$, (41)

$\sigma_{\gamma_{i}2}^{2}(k)=\frac{1}{N_{1}-1}\sum_{j=1}^{N_{1}} \left[\hat{\gamma}_{i}(k-j|k-j)-\mu_{\gamma_{i}}(k)\right]^{2}$, (42)

$\mu_{\gamma_{i}}(k)=\frac{1}{N_{1}}\sum_{j=1}^{N_{1}} \hat{\gamma}_{i}(k-j|k-j)$, (43)

*N_1_* , in Eqs. (41) ~ (43), denotes the pre-selected data window.

A threshold $\beta_{\gamma_{i}}$ is preset and$\beta_{\gamma_{i}}\succ0$, then we assume:

$H_{0}:MB_{\gamma_{i}}(k)\leq\beta_{\gamma_{i}}$, (44)

$H_{1}:MB_{\gamma_{i}}(k)\succ\beta_{\gamma_{i}}$, (45)

- If *H_1_* of *γ_i_* hold alarms, the fault indicated by parameter *γ_i_* happens.
- If *H_0_* of *γ_i_* hold alarms, the fault indicated by parameter *γ_i_* not happens.

The threshold $\beta_{\gamma_{i}}$ can be selected by computer simulations. If a smaller $\beta_{\gamma_{i}}$ is used, smaller faults can be detected, but the false alarm is expected to increase; on the other hand, if a larger $\beta_{\gamma_{i}}$ is defined, only a relatively larger fault can be detected, and thus increasing the missing alarm.
