# Supplementary material for: Fault Diagnosis for the Heat Exchanger of the Aircraft Environmental Control System Based on the Strong Tracking Filter
Source: PLoS One. 2015 Mar 30;10(3):e0122829. doi: 10.1371/journal.pone.0122829 (PMC4379147; doi:10.1371/journal.pone.0122829)
Supplement: S3 Appendix — (DOC) [file pone.0122829.s012.doc]

**Appendix C.** **Details of the Fault Detection and Diagnosis Strategy**

Details of the fault detection and diagnosis strategy are shown in S9 Fig.
